# Drug Rash With Eosinophilia and Systemic Symptoms (DRESS) Due to Multiple Anti-Epileptic Drug Hypersensitivity

**DOI:** 10.7759/cureus.65417

**Published:** 2024-07-26

**Authors:** Alyssa Roseni S Ajon, Pia Teresa A Camara

**Affiliations:** 1 Department of Adult Neurology, Center for Neurological Sciences, Quirino Memorial Medical Center, Quezon City, PHL

**Keywords:** hypersensitivity, levetiracetam, gabapentin, aromatic anticonvulsants, dress syndrome

## Abstract

Drug rash with eosinophilia and systemic symptoms (DRESS) is a rare type of hypersensitivity reaction with an incidence in the general population of one case per 10,000, which may lead to life-threatening complications with a mortality rate of 3.8% to 10%. This condition has been characterized by the following symptoms: skin rash, febrile episodes, lymph node enlargement, and involvement of internal organs, specifically the liver. Common medications associated with these reactions are aromatic anticonvulsants and antibiotics.

In this paper, we report a case of a 41-year-old female presenting with head-turning to the right and stiffening of the right upper and lower extremities, with subsequent involvement of the left upper and lower extremities secondary to a left frontal lobe glioma. She had a hypersensitivity reaction to five anticonvulsant medications and was treated with prednisone pulse therapy. This case showed that DRESS syndrome may also manifest with newer non-aromatic anticonvulsants such as levetiracetam and that steroid pulse therapy is effective in resolving the elevated blood parameters as well as skin lesions of patients with DRESS syndrome. The patient’s epilepsy responded well to gabapentin without any recurrence of seizure episodes or hypersensitivity reactions.

## Introduction

Drug rash with eosinophilia and systemic symptoms (DRESS) syndrome is a type of hypersensitivity reaction seen in 1 in 10000 cases in the general population that may lead to mortality [1]. This condition, which is especially seen in anticonvulsant medications, has the following clinical features: cutaneous eruption, febrile episodes, lymphadenopathies (usually peripheral), and damage to one or more organs, such as the liver and kidneys, which are often potentially life-threatening. The skin rash that is suggestive of DRESS syndrome includes maculopapular or generalized erythematous rashes associated with facial edema [2,3].

The exact pathogenesis of DRESS is still not clear. Bohan et al. suggested that in cases related to anti-convulsant therapy, there are three components that are considered to have an elaborate interaction resulting in DRESS: 1. A deficiency or abnormality of the epoxide hydroxylase enzyme that detoxifies the metabolites of aromatic amine anticonvulsants; 2. Associated sequential reactivation of the herpes virus family; 3. Ethnic disposition with certain human leukocyte antigen (HLA) alleles [2,4-6].

Theory of deficiency or abnormality of the epoxide hydroxylase enzyme: there are genetic polymorphisms that appear to be autosomal dominant in inheritance, causing mutations of genes that encode for drug detoxification enzymes, leading to the accumulation of drug metabolites that trigger an autoimmune response against skin and liver cells [5,7,8]. Epoxide hydroxylase detoxifies arene oxide metabolites, a product of the CYP450 system metabolism of anticonvulsants. A defect in this enzyme causes the accumulation of toxic metabolites and elicits an immunologic response [7,8].

Theory of reactivation of herpes viruses: systemic manifestations of DRESS are related to human herpes virus reactivation and host immune response, as HHV-6 was detected in the blood of approximately 60-80% of patients with DRESS in the study of Kano et al. [8].

Theory of genetic predisposition with HLA alleles: having specific HLA haplotypes may predispose an individual to developing DRESS when exposed to an inciting drug. It has been thought that the drug interacts with a particular HLA forming a complex hapten or prohaptens which is presented to naïve T cells and would subsequently trigger an immune response depending on the HLA expressed. Examples of these are HLA-A*3101 which increased the risk of Japanese patients in the study of Kashiwagi et al. for developing DRESS syndrome exposed to carbamazepine. HLA-DR3 and HLA DQ2 are, likewise, associated with an increased frequency of developing carbamazepine-induced DRESS syndrome [7-9].

The most frequently affected visceral organ is the liver, with liver abnormalities with elevated serum alanine transferases (ALT) found in approximately 70% of cases with DRESS syndrome and even up to 95% in a series of 27 patients with DRESS. The elevated liver enzymes may persist for several days after withdrawal of the offending drug [7].

Aromatic anticonvulsants such as phenobarbital and carbamazepine are the most frequently involved drugs in DRESS, and the risks for developing DRESS syndrome with specific anticonvulsant medications are varied. It has been noted in the study of Blaszczyck et al. that phenobarbital accounts for 15%, phenytoin for 13%, carbamazepine at 11%, and oxcarbazepine accounts for below 5% of the risk of incidence of DRESS syndrome [3].

In this report, we cite a 41-year-old female who satisfied the RegiScar criteria for DRESS syndrome and was exposed to five antiepileptic medications, namely: phenobarbital, carbamazepine, valproic acid, levetiracetam, and zonisamide, with cross-reactivity not entirely ruled out due to the interval of administration. A Naranjo score of 11 was computed for the patient.

## Case presentation

This is the case of a 41-year-old female who, 14 days before presentation, was admitted to a different hospital due to stiffening of the right upper extremity with versive head movement to the right, progressing to a generalized stiffening of all extremities and upward rolling of the eyeballs, subsequent loss of consciousness, and post-ictal confusion. She was managed as a case of viral encephalitis and treated with IV Acyclovir for 10 days at the previous hospital. No cranial imaging was done at the time, and the patient was discharged with a regimen of phenobarbital 60 mg/tab twice a day, which was started on the day of discharge. The patient had no prior history of allergies to food or medications or bronchial asthma. The patient had been taking the prescribed phenobarbital for 1-2 days when she started to note erythematous macular lesions over the extremities and torso without involvement of mucosal tissue at this time. The lesions were subsequently resolved with the intake of antihistamine. No consultations were done during this time, and the medication was discontinued altogether.

On the day of presentation to the emergency room, which was 11 days from the initial development of a skin rash, the patient had a recurrence of the same seizure pattern. No rashes were noted at this time, and phenobarbital was reintroduced at the same dose. The patient was observed for 24 hours for the possibility of a recurrence of the allergic reaction. No rashes or recurrence of seizures were noted.

On the fourth day of admission, she started to develop erythematous macular lesions over the arms. She was started on cetirizine (5 mg/tab) and phenobarbital was discontinued. The anticonvulsant medication was then shifted to carbamazepine 200 mg/tab twice a day. There was a decrease in erythematous rashes upon intake of antihistamine. The patient, on the eighth hospital day, was noted to have more pruritic lesions over her extremities despite daily antihistamine medications. She was referred to the Allergology service and started on hydrocortisone 100 mg/IV, and cetirizine was continued. Carbamazepine was discontinued and was observed for 24 hours. There was a slight improvement in the pruritus of the patient upon starting hydrocortisone.

As part of her workup, she underwent an electroencephalogram, which showed active epileptiform discharges. She was then started on Valproic Acid 500 mg/tablet twice a day due to the EEG findings, although there was no recurrence of frank seizures.

**Figure 1:**
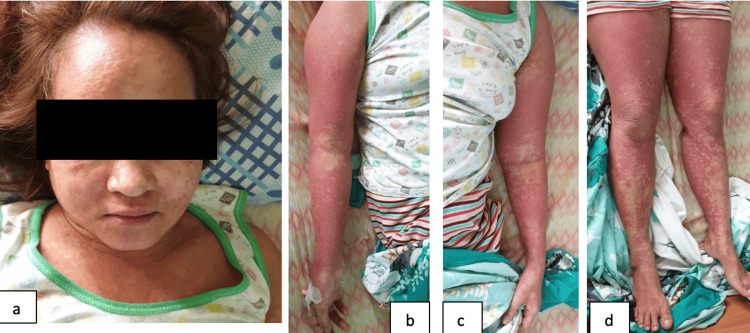
1a-1d shows morbilliform, coalescing rash over the face, torso and legs on the 15th hospital day

On the 12th day of hospitalization, the patient developed facial edema as well as progression of the morbilliform, erythematous macular reactions that involved all her extremities as well as her back and torso. There was no mucosal involvement at this time. Valproic acid was discontinued and was started on levetiracetam 500 mg/tab twice a day. However, she developed a high-grade fever, cervical lymphadenopathies, and an increase in facial edema. Repeat laboratories showed eosinophilia of 21%, an aspartate aminotransferase of 127.38 U/L, and an alanine aminotransferase of 186.23 U/L. All anti-epilepsy medications were discontinued on the 15th hospital day without any recurrence of seizures. Her facial edema and rashes were still present, as seen in Figure 1a-1d. The allergology and dermatology services both agreed to start the patient on methylprednisolone 1 mg/kg/day for three days, which was tapered for two months with oral prednisone dosing. After the initial methylprednisolone dose, improvements in the lesions and blood parameters of the patient were noted. She was discharged on the 19th day of admission without any anti-epileptic medications.

The patient was then seen at the outpatient service two weeks after discharge, with her latest EEG result still showing epileptiform discharges. Zonisamide 25 mg/tab twice a day was started as an outpatient but developed erythematous rashes over the right upper extremity and was immediately discontinued. No progression of the rashes was noted.

Further work-up for the etiology of the seizure was done as an outpatient, including a cranial MRI with contrast, which revealed a parasagittal mass over the left frontal lobe. She underwent a craniotomy and the excision of a tumor. Histopathology reported a low-grade glioma. She then had a recurrence of her seizure described as right upper extremity without versive head movement, progressing to a generalized stiffening of all extremities and upward rolling of eyeballs, and subsequent loss of consciousness and post-ictal confusion lasting for approximately 2 minutes. Gabapentin 300 mg/tab every 8 hours was started. No recurrence of seizures was noted, and no rashes developed until her discharge after 23 days. She was maintained on Gabapentin 300 mg/tab every 8 hours and has been seizure-free since.

**Figure 2:**
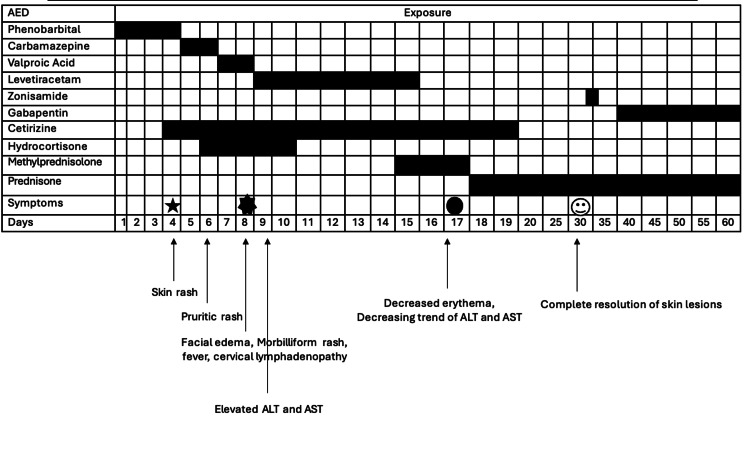
Timeline diagram with chronologic sequence of clinical symptoms (x-axis) and medication use (y-axis)

Figure 2 summarizes the timeline of anti-epileptic medications given, the chronology of the development of symptoms, and the day of resolution of symptoms. 

## Discussion

Drug reaction with eosinophilia and systemic symptoms (DRESS) is a severe adverse drug-induced reaction with an estimated incidence ranging from 1 in 1000 to 1 in 10,000 drug exposures [3]. Several medications have been implicated in the development of DRESS, specifically aromatic anticonvulsants, especially phenytoin, carbamazepine, phenobarbital, and sulfonamides such as dapsone and sulfasalazine [7]. Bocquet et al. described the syndrome as including a severe skin eruption, fever, hematologic abnormalities (eosinophilia or atypical lymphocytes), and internal organ involvement. In the literature, hepatitis is reported to be common and occurs in up to 90% of cases, which is consistent with our case. Renal (9%) or pulmonary involvement (5%) is less commonly seen. These manifestations, including mild mucosal involvement and eosinophilia, differentiate DRESS from Steven-Johnson syndrome (SJS) [4]. SJS would have erythematous macules with purpuric lesions progressing to vesicles or bullae and sloughing with more than 90% mucosal involvement and lymphopenia [4].

Pharmacogenetic variation in drug biotransformation has been suggested to be one of the causes of these undesired side effects. It has been thought that DRESS involves a deficiency or abnormality of the epoxide hydroxylase enzyme that detoxifies the metabolites of aromatic amine anticonvulsants. The reactivation of herpes-type viruses and ethnic predisposition to certain human leukocyte antigen subtypes were also considered to play a role in its development [3]. Through the metabolism of AEDs, there can be an accumulation of toxic metabolites that can directly cause cell death or, as pro haptens, bind to T-cells, which will invoke an immune response [5-8].

DRESS syndrome’s treatment of choice employs intravenous administration of steroids, specifically prednisone at a dose of 1-1.5 mg/kg or any equivalent corticosteroid. This is maintained until complete resolution of the rash is achieved and is then tapered slowly. Alternative treatments include the use of immunoglobulins to compensate for the immunosuppression that accompanies the syndrome and also for their anti-inflammatory effect [10].

The patient developed lesions on the fourth hospital day, which progressed with every antiepileptic medication given to the patient. The symptoms started to improve on the second day of administration of methylprednisone pulse therapy, and complete resolution of the lesions was seen after 20 days of initiation. The patient fulfilled all four criteria required to make the diagnosis of DRESS as per the RegiSCAR criteria (Table 1).

**Table 1:** Inclusion criteria for potential cases of drug reactions with eosinophilia and systemic symptoms published by RegiSCAR * Three of these four criteria are required for diagnosis

RegiSCAR Criteria	Patient
Hospitalization	1
Reaction suspected to be drug-related	1
Acute rash	1
Fever > 38 C*	1
Enlarged lymph nodes at a minimum of two sites*	1
Involvement of at least one internal organ*	1
Blood count abnormalities*	1
Lymphocytes above or below normal limits	-
Eosinophils above the laboratory limits	-
Platelets below the laboratory limits	-

## Conclusions

In conclusion, drug rash with eosinophilia and systemic symptoms still lacks a definitive pathogenesis but has been found to be treated effectively by immediate withdrawal of the offending agent and moderate-to-high-dose corticosteroid therapy. Clinicians following the standard of therapy, especially for AED, in places lacking alternative medications for the treatment of epilepsy should be wary of inducing hypersensitivity reactions even in patients without a prior history of allergy. A high index of suspicion for the development of DRESS syndrome shall be employed in patients given anticonvulsant therapy. Further, the presented case has shown that gabapentin seems to be an effective alternative for seizure control in patients who have a history of DRESS syndrome.
